# The impact of a meta‐analysis on orthopaedic surgeons' practice with regards to vancomycin graft pre‐soaking in anterior cruciate ligament reconstruction: A paper questionnaire survey study

**DOI:** 10.1002/hsr2.1009

**Published:** 2022-12-25

**Authors:** Kenan Kuršumović, Charalambos Panayiotou Charalambous

**Affiliations:** ^1^ Department of Orthopaedics Blackpool Victoria Hospital Blackpool UK; ^2^ Department of Orthopaedics, School of Medicine University of Central Lancashire Preston UK

**Keywords:** English: anterior cruciate ligament reconstruction, infection, knee, vancomycin

## Abstract

**Background and Aims:**

Understanding the impact of orthopaedic scientific research is vital in identifying facilitators and barriers to its implementation in clinical practice. A meta‐analysis was carried out which showed that presoaking hamstring (HT) autografts in vancomycin was associated with a 10‐fold reduction in infection rate in anterior cruciate ligament (ACL) reconstruction. Our aim was to determine the practice of orthopaedic surgeons with regards to vancomycin presoaking and explore whether they would adopt the findings of this meta‐analysis.

**Methods:**

A paper questionnaire survey was administered to attendees of an annual EFORT podium presentation of the meta‐analysis findings. Descriptive statistics were used to summarize the characteristics of respondents and their responses.

**Results:**

A total of 29 senior surgeons/subspecialists performing a median of 40 ACL reconstructions per year completed the survey of whom 7 (24.1%) had encountered an ACL graft infection in the previous 2 years and 14 (48.3%) in the previous 5 years. Only 3 (10.3%) presoaked the ACL graft with an antibiotic. About 1/4 of those who up to then did not pre‐soak the graft (6/26, 23.1%) would consider changing their practice to pre‐soaking with vancomycin, with similar findings (5/20, 25.0%) in those that used a HT autograft as their first choice.

**Conclusions:**

Orthopaedic surgeons are receptive to the findings of a meta‐analysis reporting on the effectiveness of vancomycin graft presoaking in ACL reconstruction, which can thus have a substantial impact upon clinical care. Addressing concerns about vancomycin induced graft toxicity and comparing the pre‐soaking effect to that of specific intravenous antibiotic regimens may further enhance the uptake of this practice.

## 1 BACKGROUND

**Figure 1:**
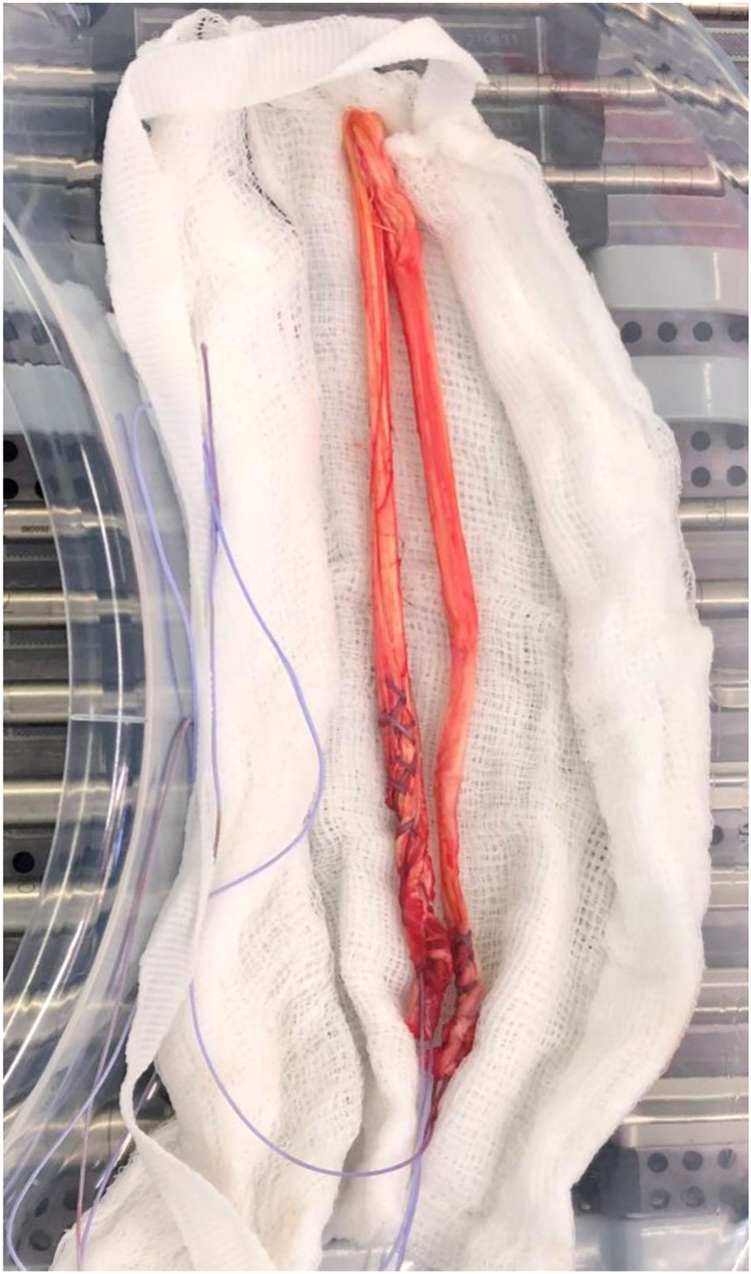
The hamstring graft to be used for ACL reconstruction is wrapped in a vancomycin‐soaked gauze swab to minimize the risk of infection. ACL, anterior cruciate ligament.

Septic arthritis following anterior cruciate ligament (ACL) reconstruction is a devastating complication which usually requires further surgery and may lead to graft failure and poor knee function.^1^ Our team performed a meta‐analysis to examine the role of vancomycin graft presoaking (Figure 1) in reducing infection bacterial rates in ACL reconstruction. We showed that hamstring (HT) autografts were associated with a statistically significant higher infection rate as compared to bone‐patellar tendon‐bone (BPTB) autografts and allografts. Furthermore, it was shown that presoaking HT autografts in vancomycin was associated with a 10‐fold reduction in infection rates.^2^

The process of implementing findings of orthopaedic research in clinical practice involves adequate communication of research findings to improve the knowledge of orthopaedic surgeons.^3^, ^4^, ^5^, ^6^ Surgeons may then use evidence from such research to implement changes in their clinical practice. National and international meetings of general orthopaedic and specialist societies provide an opportunity whereby research findings are presented at an early stage, before the full peer reviewed publication. The annual EFORT congress is such a venue with a large international audience of attendees. Hence, to communicate our results and encourage a change in clinical practice, the findings of our meta‐analysis were presented as a podium presentation at the 18th EFORT annual congress in Vienna, Austria, in May 2017. The results of our meta‐analysis study were subsequently published in a peer review journal.^2^

Understanding the impact of research findings upon potential change in clinical practice of the orthopaedic community is valuable in the process of promoting its implementation. Facilitators and barriers to acceptance of new research findings are thus identified and explored.

Our aim was to determine the practice of orthopaedic surgeons who carry out ACL reconstructions with regards to vancomycin presoaking and explore whether they would adopt the findings of this meta‐analysis.

## 2 MATERIALS AND METHODS

A questionnaire was constructed for the purposes of this study, consisting of 10 questions. This was administered to participants of the 18th EFORT annual congress attending a podium presentation by the first author of the results of a meta‐analysis evaluating the role of vancomycin graft presoaking upon infection rates of ACL reconstruction surgery.^2^ Printed copies of the questionnaire were placed on all the seats for the attendees on the free paper session on ACL outcomes on the first day of the congress (May 31, 2017) and they were referred to during the presentation of the paper. They were then collected at the end of the session and analysed using Microsoft Excel 2007.

**Table 1:** Demographics of respondents at the survey of 18th EFORT annual congress

Level of surgical practice	Senior surgeons/subspecialists—29
	Fellows—0
	Residents/Trainees—5
Surgeon's main subspecialty interest(s)	Sports surgery—17 (58.6%)
	Knee surgery—22 (75.9%)
	Other (e.g., shoulder)—8 (27.6%)
Region of practice	Europe—12 (41.4%)
	Middle East—7 (24.1%)
	Australia/New Zealand—3 (10.3%)
	South Asia (India/Pakistan)—3 (10.3%)
	East Asia (Philippines/Indonesia)—3 (10.3%)
	South America (Colombia)—1 (3.4%)
Years in practice	Mean 17.2
	Median 17.0 (range: 2–45) years
Annual ACL reconstruction volume	Mean 58.3
	Median 40.0 (range: 5–250)
Main graft of choice(s) for a primary ACL reconstruction	Hamstring tendon autograft—23 (79.3%)
	Bone patellar tendon bone autograft—9 (31.0%)
	Quadriceps tendon autograft—1 (3.4%)
	Allograft—0 (0.0%)
	Synthetic—1 (3.4%)^a^
Cases of intra‐articular infections following primary ACL reconstruction previously treated by respondent	In last 2 years:
	Mean 1.0
	Median 0.0 (range: 0–20)
	In last 5 years:
	Mean 2.8
	Median 2.0 (range: 0–60)

Abbreviation: ACL, anterior cruciate ligament.

^a^ In over 40s only.

## 3 RESULTS

Of the respondents, 29 senior surgeons/subspecialists performing a median of 40 ACLs per year completed the survey of whom 7 (24.1%) had encountered an ACL graft infection in the previous 2 years and 14 (48.3%) in the previous 5 years (Table 1). Only 3 (10.3%) presoaked the ACL graft with an antibiotic. About 1/4 of those not presoaking the graft (6/26, 23.1%) would consider changing their practice to presoaking with vancomycin, with similar findings (5/20, 25.0%) in those that used a HT autograft as their first choice (Table 2). One of the surgeons that did not presoak their ACL grafts cited concern about possible toxicity to the graft by vancomycin as the reason for not considering a change in practice; another responder felt that the current intravenous preoperative and postoperative prophylactic antibiotic regimen was sufficient at addressing the infection risk. 

**Table 2:** Respondent's practice in relation to graft presoaking and impact of the findings of the meta‐analysis on change in practice

Current ACL graft practice with regards to antibiotic presoaking	3/29 (10.3%) senior surgeons pre‐soak the ACL graft Vancomycin—2 Gentamicin—1 3/23 (13.0%) of senior surgeons that regularly use HT autografts presoak the ACL graft
Would respondent consider changing practice to vancomycin presoaking of ACL grafts, in view of the presented meta‐analysis findings?	6/26 (23.1%) senior surgeons that did not pre‐soak the ACL graft would consider changing practice to presoaking with vancomycin. 5/20 (25.0%) senior surgeons that used HT autografts regularly and that did not presoak the grafts, would consider changing practice to presoaking with vancomycin. 1 surgeon using gentamicin for pre‐soaking did not respond about switching to vancomycin.

Abbreviation: ACL, anterior cruciate ligament.

## 4 DISCUSSION

This study has shown that a meta‐analysis assessing the role of vancomycin graft presoaking in ACL reconstruction can have a substantial impact on clinical practice, with a considerable proportion of orthopaedic surgeons willing to consider adopting this approach.

Septic arthritis following ACL reconstruction is a devastating complication which usually requires further surgery and may lead to graft failure and poor knee function. Our team performed a meta‐analysis to examine the role of vancomycin graft presoaking in reducing infection rates in ACL reconstruction. This showed that HT autografts were associated with a higher infection rate as compared to BPTB autografts and allografts, and that presoaking HT autografts in vancomycin reduced infection rates by about a 10‐fold. Communicating these findings to the orthopaedic community is vital, hence our findings were presented at the 2017 EFORT annual congress as a podium presentation.

Our results suggest that our meta‐analysis had a substantial impact on orthopaedic practice as assessed by a survey of those attending the presentation at the EFORT congress, with about one in four of respondents reporting that they would consider applying vancomycin graft presoaking in their ACL reconstruction practice.

Knowledge and education gaps have extensively been cited in previous studies examining barriers to the implementation of medical knowledge into practice.^3^, ^4^, ^5^, ^6^ However, real concerns about emerging medical technologies and the benefit they add to existing practice may also be involved. In relation to this, one respondent reported concerns about vancomycin induced graft toxicity, whereas one questioned the value of a presoaking regimen to the already applied intravenous antibiotics. Understanding such concerns and responding to them through existent evidence, or through further work to fill knowledge gaps, may further facilitate the impact of scientific research.

Communication of findings through presentations in national and international meetings of general orthopaedic or more specialist societies may be of value. It may also allow faster uptake of findings, as there is often a lag between research findings becoming available and appearing in a peer‐reviewed journal.^7^, ^8^ It has previously been shown that of those EFORT congress presentations going on to full journal articles 32% were published within 1 year of the EFORT congress and 63% within 2 years.^7^ Our results also suggest that a podium presentation at the annual EFORT congress can achieve communication and uptake of its findings. Furthermore, this study suggests that the EFORT congress is a good venue for assessing impact on clinical practice uptake of research findings, and consideration should be made as whether this is more regularly implemented, as it would be an important feedback tool to presenters and their research team.

Limitations of this study include the fact that the response rate cannot be calculated. Furthermore, a willingness of a large proportion of participants to consider changing their practice does not imply an actual change in practice as other institutional and resource barriers to change may exist.^9^, ^10^, ^11^ However, a willingness to consider a change in practice is the first step for any implementation of new evidence.

## 5 CONCLUSIONS

Our findings suggest that the findings of a meta‐analysis demonstrating the effectiveness of vancomycin presoaking in reducing infection rates in ACL reconstruction can have a substantial impact on the clinical practice of orthopaedic surgeons. Addressing concerns of vancomycin induced graft toxicity and further exploring the additive protective effect of vancomycin over specific peril‐operative intravenous antibiotic regimens may further encourage the uptake of this process.

## AUTHOR CONTRIBUTIONS

**Kenan Kuršumović**: conceptualization; data curation; formal analysis; investigation; methodology; resources; writing – original draft; writing – review & editing. **Charalambos Panayiotou Charalambous**: conceptualization; data curation; formal analysis; methodology; supervision; writing – original draft. All authors have read and approved the final version of the manuscript, CP Charalambous had full access to all the data in this study and take complete responsibility for the integrity of the data and the accuracy of the data analysis.

## CONFLICTS OF INTEREST

The authors declare no conflicts of interest.

## TRANSPARENCY STATEMENT

The lead author Charalambos Panayiotou Charalambous affirms that this manuscript is an honest, accurate, and transparent account of the study being reported; that no important aspects of the study have been omitted; and that any discrepancies from the study as planned (and, if relevant, registered) have been explained.

## Data Availability

Data available on request from the authors.
